# A long term time lapse microscopy technique for Arabidopsis roots

**DOI:** 10.3389/fpls.2025.1601397

**Published:** 2025-06-09

**Authors:** Laura R. Lee, Ramin Rahni, Kenneth D. Birnbaum

**Affiliations:** Center for Genomics and Systems Biology, Department of Biology, New York University, New York, United States

**Keywords:** microscopy, time lapse microscopy, root development, light sheet microscope, regeneration

## Abstract

Time lapse microscopy is a transformative technique for plant cell and developmental biology. Light sheet microscopy, which manipulates the amount of light a sample is exposed to in order to minimize phototoxicity and maximize signal intensity, is an increasingly popular tool for time lapse imaging. However, many light sheet imaging systems are not designed with the unique properties of plant samples in mind. Recent advances have decreased the cost and increased the technical accessibility of light sheet microscopy, but plant samples still require special preparation to be compatible with these new systems. Here, we apply a novel light sheet microscopy system to regenerating Arabidopsis roots damaged via laser ablation. To adapt this system for Arabidopsis roots we establish a new protocol for sample mounting, as well as an automated root tip tracking system that requires no additional proprietary software. The methods presented here can be used to increase researcher access to long-term time-lapse imaging in Arabidopsis biology.

## Introduction

Fluorescent microscopy of live samples is a fundamental technique in plant biology. Visualization of plant cells and proteins has led to discoveries such as new cell fate specifying mechanisms (Dong et al., 2009) and hormone enrichment domains regulating organ growth (Müller and Sheen, 2008; Efroni et al., 2016). Time lapse microscopy, in which successive microscopic images of a sample are taken over time, allows for the observation of dynamic processes including how cells or organs respond to stimuli (Toyota et al., 2018) and which cell types contribute to organ growth (Li et al., 2024). Confocal microscopy allows for excellent imaging resolution and acquisition of three dimensional information, but has limited ability to image deep within a tissue (Combs and Shroff, 2017) and can require laser power that causes phototoxicity when imaging fluorophores excited with short laser wavelengths (Combs and Shroff, 2017). Further, imaging an entire organ via confocal microscopy can take several minutes per fluorophore depending on the size of the organ and the model and configuration of microscope used, and this limits the time resolution that can be achieved during time lapse imaging (Combs and Shroff, 2017). Light sheet microscopy provides a similar ability to image multiple fluorophores in three dimensions, but it also solves the problems of limited light penetration, phototoxicity, and poor time resolution (Power and Huisken, 2017). As such, it is an increasingly popular microscopy approach in plant biology, particularly for imaging roots (Candeo et al., 2017; von Wangenheim et al., 2017a, 2017b; Ovečka et al., 2022).

Confocal microscopes manipulate the aperture of the objective in order to allow only a small plane of light, a couple of microns in the z dimension, of an illuminated sample to be imaged at once (Elliott, 2020). This provides high resolution but results in considerable light loss (North, 2006). Light sheet imaging solves this problem by manipulating the laser emission to illuminate a single plane of a sample at once, making it possible to image a single plane without limiting the light that passes through the objective (Hobson et al., 2022). This technique has been applied to plant roots with exciting results, leading for example to the identification of cell cycle regulated SHORTROOT kinetics (Winter et al., 2024), as well as the coordination between cell cycle and cell identity changes during root tip regeneration (Lee et al., 2025). However, they are not suitable for imaging all tissue types because samples must be embedded in gel or liquid media (Ovečka et al., 2018), meaning a free-standing light sheet microscope may not be appropriate for all plant biology labs. However, most light sheet systems were not developed with the unique characteristics of plant organs in mind. For example, plant roots grow rapidly and so will frequently move out of the field of view of the objective and will require abundant space within the sample chamber to prevent stress. This creates challenges for both sample tracking and sample mounting. We address both of these issues in this methods paper.

Here, we present a protocol for long-term time-lapse imaging of plant roots using the TILT light sheet imaging system from Mizar, which is a commercially available microscope add-on that can be used with an existing confocal microscope. A major advantage of this system is that it can be adopted by plant biology labs that work with a variety of organs because it is relatively inexpensive to implement, and it does not require highly specialized optical configurations. Therefore, this approach may enable more plant biology labs that already use confocal microscopy to additionally take advantage of light sheet imaging. While some of the benefits of this system are that it is lower cost and has a smaller footprint than traditional light sheet microscopes, it presents a new challenge based on the required flat orientation of the sample, particularly when performing time lapse imaging of organs such as roots that are gravitropic. Below we detail the preparation of Arabidopsis roots such that they can be successfully imaged in this system. Then we demonstrate the utility of the system with a 24-hour time lapse of a regenerating root expressing three fluorescent reporters with ten-minute time resolution and full z depth.

## Materials and equipment

The required materials and equipment are detailed below.

- Arabidopsis plants, optionally expressing a reporter for quiescent center (QC) or columella cells if tracking is desired.
- Computer running Windows 10 with an Intel^®^ Core™ i9-9820X 3.3 GHz processor, an NVIDIA GeForce RTX 2080 Ti graphics card, 128 GB RAM for time lapse imaging analysis. The custom workstation we used was designed before 2020, so alternative and more appropriate models may now be available.
- Mizar TILT system.
- A microscope compatible with the Mizar TILT. We used a Leica model Dmi8 low (10x) and high (40x) objectives. The lower power objective is most useful for sample finding, while the higher power objective is ideal for imaging Arabidopsis roots. The NA of the 40x objective should be high. We used a HC PL APO 40x/1,1 W CORR CS2 (Leica #11506425).
- A computer workstation appropriate for running the microscopes via VisiView software. Our workstation has the following configuration: Intel Core i5-13600 2.70G 24MB 14 cores 65W CPU, 64GB (2x32GB) DDR5–4800 UDIMM ECC Memory, NVIDIA RTX A2000–12 GB GDDR6 4mDP Graphics, HP 512GB PCIe-4x4–2280 Value M.2 Solid State Drive, 8TB 7200RPM SATA 3.5in Enterprise, and a Windows 10 operating system.
- 1 Well chambered cover glass with optically clear sides (CellVis #C1-1.5H-N).
- Low melt agarose (VWR #89125-532).
- MS salts (Sigma #M5524).
- 0.45 micron syringe filter (VWR #76479-042).
- 50 mL Syringe (BD #309653).
- Autoclave or ethanol sterilized forceps, pointed (Fisher #12-000-124) and flat (Fisher #16-100-116), 2 each.
- Sterile ultrathin scalpel blades (Fisher #13-812-230).
- If generating large files, Imaris license.
- If generating small files, FIJI with correct 3d drift plugin and trackmate plugin.
- 1 mL pipette.
- 1 mL Filter tips.
- Sterile hood.
- Autoclave.
- Water bath.
- Sterile box, like a phytatray (Millipore Sigma P5929) or an autoclaved pipette tip box.
- Sterile surface such as an empty petri dish.
- Large file storage options such as a solid-state drive or cloud storage environment.

## Methods

### Sample preparation

#### Plant growth

Grow plants vertically on ½ MS as described in (Lee et al., 2025) for 6 days. Grow many more seedlings than is required for imaging. Although only one root will be imaged per time lapse, it is possible some roots will become damaged during mounting. In addition, some roots may not be in an ideal orientation for imaging after mounting and preparing excess roots increases the likelihood of obtaining an imageable sample. Finally, for longer-term imaging experiments, it is necessary to mount two roots close together such that one can use the other as a guide to ensure a desirable growth direction is achieved.

#### Mounting media, tools and preparation

1 day prior to mounting, prepare mounting media. Prepare 25 mL of media per imaging experiment. Stratify Arabidopsis seeds at 4°C for 2 days, sterilized, placed on agar plates containing ½ Murashige and Skoog salts (Sigma M5524), 0.5% sucrose, and grow vertically in chambers set to 23°C and a 16h light/8h dark cycle (80-90 μmol m-2 s-1). Replace agar in the ½ MS media with low melt agarose such that the final concentration is 2% w/v. The media needs to be filtered so that it is optically clear for use with light sheet imaging. Therefore, the media can be filter-sterilized using a 0.22 micron syringe filter rather than autoclaving. Media can then be held at 4˚C until needed. To re-melt, place the falcon tube containing solidified media into a beaker containing miliQ water, partially unscrew the lid of the falcon tube, and microwave on 50% power for 30 second intervals until the media has fully melted. Reseal the falcon tube and gently invert to ensure the media is homogenous. Then hold in a constant temperature water bath at 30˚C until needed.

To prepare media blankets obtain a fresh chambered cover glass. Open it in a sterile environment like a tissue culture hood or work under a flame. Using a 1 mL pipette with sterile filter tips, add 5 mL of media to the chambered cover glass. Pipette off any bubbles from the surface. Close the chambered cover glass and allow the media to solidify in the tissue culture hood. Transfer the chambered cover glass to a closed sterile container, which will prevent contamination and desiccation, and store at 4˚C for at least 24 hours and up to one week. It is easier to manipulate the fully chilled blanket than one that is freshly poured. Prepare two or three of these blankets per imaging session to ensure sufficient material is available in case a blanket becomes damaged during the next step.

**Figure 1:**
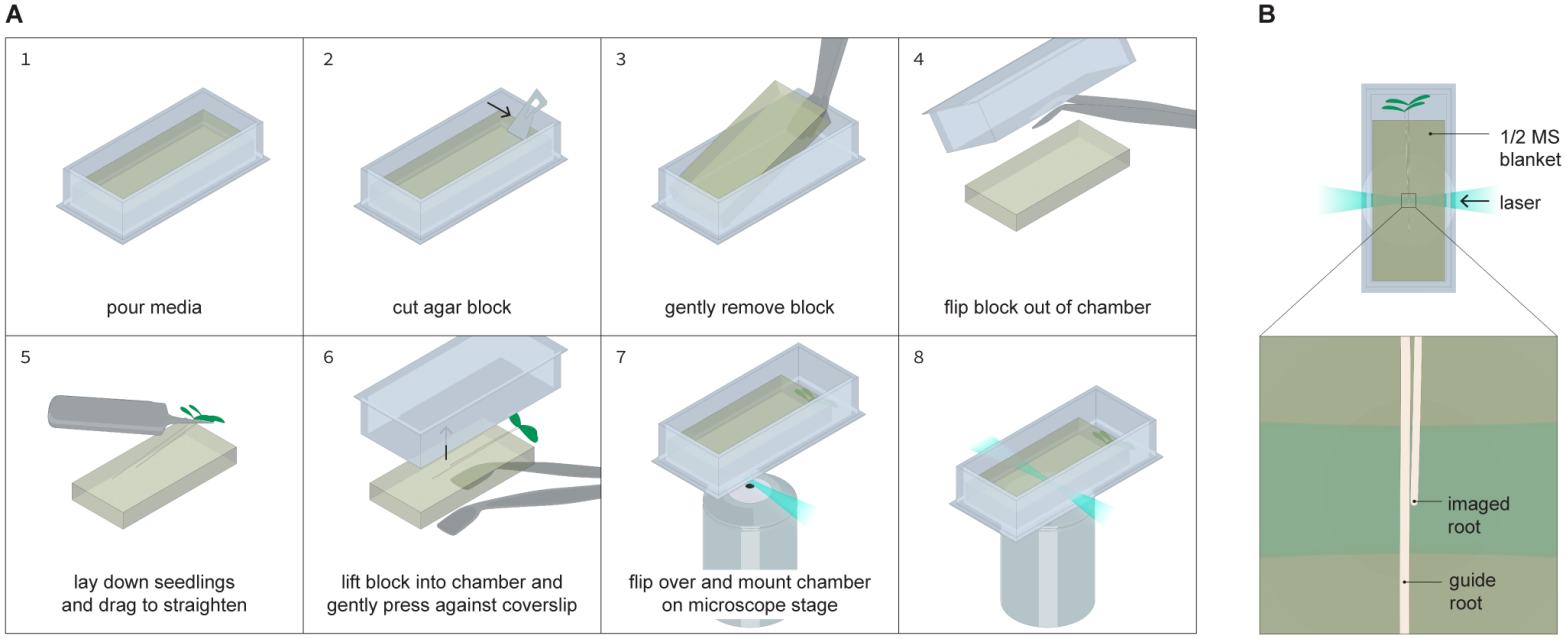
Seedling mounting procedure. **(A)** Step by step process for positioning seedlings within an imaging chambered cover glass that is compatible with the TILT system. A media blanket is cast in an imaging chambered cover glass, after which the blanket is removed from the chambered cover glass, seedlings are placed on the blanket, and then the blanket and seedlings are placed together in a fresh chambered cover glass such that the seedlings’ roots are sandwiched between the blanket and the coverslip at the bottom of the chambered cover glass. **(B)** Overhead view of the fully assembled sample, showing how two seedlings are positioned together within the chambered cover glass with the root tips staggered. One root guides the growth of the other, with the imaged root being positioned closer to the side of the chambered cover glass the laser enters through.

An alternative protocol for media blanket preparation is to cast media in a petri dish and then cut out blankets of an appropriate size using a sterile blade. It would then be possible to fit this blanket into the chambered cover glass as shown in **Figure 1A** beginning in panel 5. This would be desirable if chambered cover glasses are in limited supply but may also require some trial and error in producing blankets of an appropriate size to fit into the chambered cover glass without causing optical distortions.

#### Sample mounting

When ready to mount seedlings as illustrated in **Figure 1**, work in a sterile environment and remove the blanket from the chambered cover glass it was cast in. For this step, gather sterile flat forceps, an empty petri dish, 6 dpg seedlings, and unused, sterile scalpel blades. Hold an aliquot of ½ MS + 2% low melt agarose in a constant temperature water bath at 30˚C in a liquid state. Remove the cover of the chambered cover glass and use a fresh scalpel to make a cut at a 90 degree angle to the long wall of the chambered cover glass at about ⅕ of the distance between the two shorter walls. Keeping the blade as flat against the sidewalls as possible, run the scalpel along each wall of the chambered cover glass to loosen the blanket. Use flat forceps to remove the smaller piece (it’s ok if this piece falls apart). Then, carefully slide a pair of flat, preferably canted forceps underneath the blanket to slowly lift it out of the chambered cover glass. Ideally the blanket should lift out in one piece. This may take a few attempts, which is why it is best practice to prepare more than one blanket per imaging experiment.

Once the blanket is freed from the chambered cover glass, set it down with the forceps underneath it in a sterile petri dish. Use sterile pointed forceps to gently lift two to three Arabidopsis seedlings and place them on top of the blanket by gliding them along the surface of the blanket such that the roots are parallel with the long wall of the blanket and the shoot is hanging off the short edge of the blanket. Make sure the roots are staggered such that one root tip is a few millimeters lower than the other, and the two roots are touching. The root tip placed lower is the guide root and the higher one is the tracked root. Ideally, the seedlings should be placed slightly off center along the short edge of the chambered cover glass (closer to one of the long walls than the other). The tracked root should also be closer to the nearest long wall.

Once seedlings are placed on the blanket, take a fresh chambered cover glass. Flip it over so that the cover slip is facing up and lower it over the seedlings and blanket so that the seedlings will be sandwiched between the blanket and the cover slip. Use the flat forceps to carefully raise the blanket until it contacts the cover slip, then flip the chambered cover glass over. There will be small bubbles between the blanket and the cover slip, but those should diffuse away overnight. If there are any gaps between the blanket and the side walls of the chambered cover glass, carefully add 30˚C media 250 µL at a time until no gaps remain. Make sure the aerial tissue does not become covered by media. When the newly added media solidifies, cover the chambered cover glass, and secure it to a flat surface that can be placed vertically in a growth chamber, such as a 96 well plate rack. Be sure not to make any marks or smudges on the long walls of the chambered cover glass. Place the chambered cover glass vertically in a growth chamber for 24 hours to allow the seedlings to recover from any transfer stress.

### Time lapse acquisition

#### Parameter selection

Acquisition parameters should be carefully selected based on the goals of the imaging experiment. For instance, an experiment tracking cell divisions in the stele would ideally be performed with a maximum of 15 minutes between time points to make it possible to track small and tightly crowded cells. As an example, for tracking cell cycle behavior with PlaCCI (Lee et al., 2025), we used the following parameters: Time points were acquired every ten minutes, slices were acquired every 1.5 microns, stacks were acquired in four channels, with channels switching between stacks rather than slices. Time resolution parameters were chosen to facilitate tracking of nuclei over divisions, which can happen quickly, while also considering final file size. We found ten minutes allowed for nuclei tracking without producing overly large files. Slice thickness was determined with a similar consideration such that we could ensure all nuclei in the root were imaged without producing files that were unreasonably large.

The full z depth of the growing root can be captured with light sheet imaging, so the z acquisition range was set from the coverslip to just beyond the far side of the root. This accounts for potential drift of the root in the z dimension during the time lapse at the cost of file size. We have found a slightly larger file is a good tradeoff to ensure the entire z dimension of the root is captured even when the sample drifts.

Laser intensity and exposure times should be optimized per reporter line and should balance the needs of signal detection with photobleaching, although in our tests photobleaching did not occur. For a relatively dim reporter, such as PlaCCI, we found laser power of 50% and exposure times of 200 ms were sufficient to produce images with high signal to noise ratio without leading to photobleaching.

#### Sheet positioning

There is a detailed description of positioning the light sheet using the TILT system available from Mizar (https://www.mizarimaging.com/wp-content/uploads/2020/04/Tilt-Manual.pdf). We found some additional steps led to improved overall performance for roots, which move much more in the x and y dimensions over the course of long-term time lapse than most live samples.

**Figure 2:**
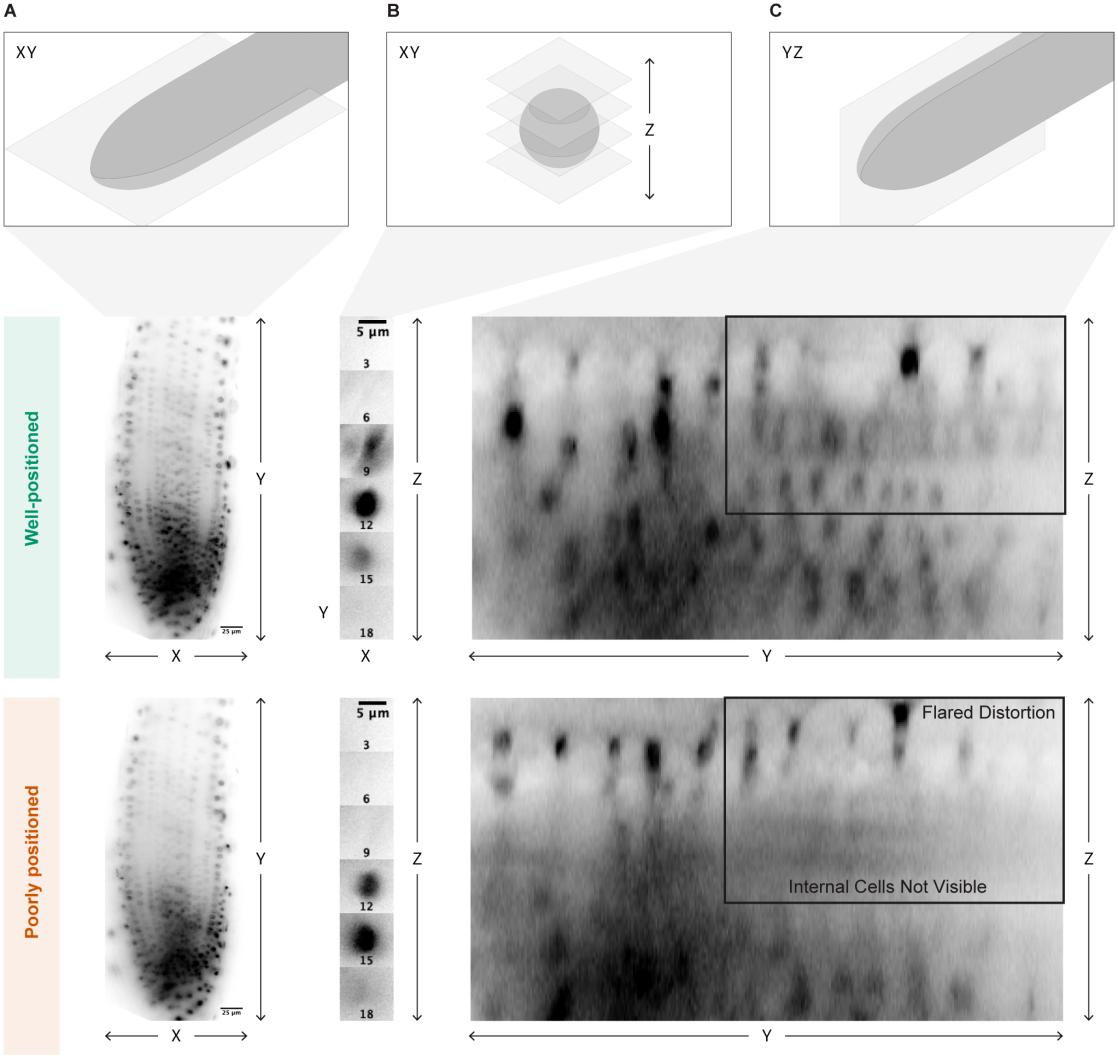
Optimal light sheet positioning. Visual indicators of optimal light sheet placement are shown. **(A)** The placement should be analyzed by bright and even illumination of the sample in the xy dimension. **(B)** Care should also be taken to look at individual bright points in the xy dimension, one nucleus as shown here, and then scrolling through the z dimension to ensure that point is brightly illuminated once. **(C)** Finally, the sample should also be examined in the yz dimension to check for flared distortions of individual points even illumination in all z layers. The middle panel shows a properly aligned sheet, while the lower panel shows a poorly positioned sheet. The poor alignment is only obvious in the yz dimension, where it is clear that some nuclear files are not clearly illuminated and there is visual distortion of individual nuclei.

We have found that slight imperfections in the position of the light sheet that might not be noticeable with a sample that moves less than a root can lead to uneven illumination during time lapse acquisition as the root grows. It can be difficult to determine whether the sheet is perfectly positioned by looking only in the x and y dimensions. However, we find that examination of an orthogonal slice of the root along the y and z dimensions (**Figure 2**) provides a good indication of sheet positioning. When perfectly positioned, the sheet will illuminate the entire z dimension of the root, while an imperfectly positioned sheet will cause portions of the root to appear blurry. A straightforward way to obtain this view is to save a z-stack of the root, open it in FIJI, and select the “Orthogonal Views” option to explore the image in the y and z dimensions. If the sheet is not perfectly positioned, fine tune it, take another z-stack, and check the orthogonal views again.

#### Automated root tip tracking

**Figure 3:**
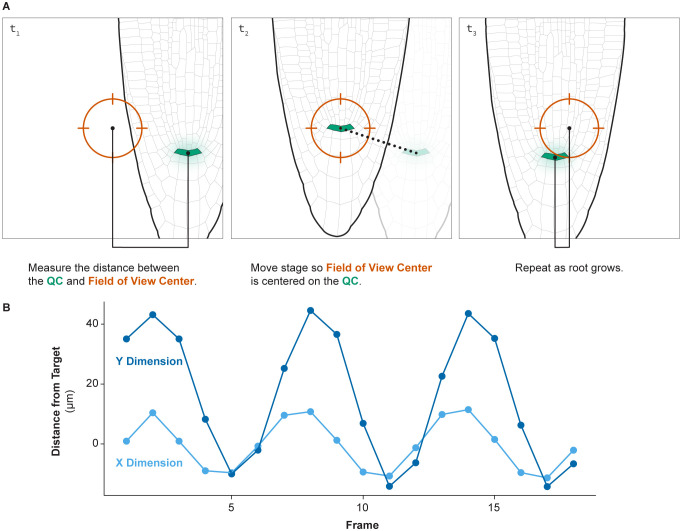
Root tip tracking. **(A)** An illustration of how the root tip tracking algorithm functions. The script measures the distance between the center of the field of view and a bright marker, in this case the QC. The script then moves the stage such that the QC is in the center of the field of view. This process then repeats each imaging cycle. **(B)** The distance of the QC to the center of the field of view over time during a 24-hour time lapse of a growing root demonstrates the successful ability of this approach to consistently track a moving root tip over time.

To run the tracking algorithm, illustrated conceptually in **Figure 3**, it is necessary to run two macros simultaneously: one in FIJI and one in VisiView. The FIJI macro will identify the center of mass of a reporter marking a small group of cells such as the QC or columella. The VisiView macro identifies the distance between the reporter’s center of mass and the center of the field of view. Then it will move the stage to move the reporter into the center of the field of view.

After specifying the acquisition parameters in the VisiView graphic user interface, initiate the time lapse by running the VisiView macro.

After the second acquisition is complete, run the FIJI macro. Upon launch, FIJI will produce dialogue boxes allowing the user to specify the run directory, the pause time between acquisitions, and the channel to perform the operation on. The FIJI macros will then write out a text file containing the (x, y) position of the center of mass, which the VisiView macro will then read and use to adjust the stage position. If the reporter used for tracking remains in the field of view during the entirety of the acquisition period, it will not be necessary to manually correct the stage position.

If the portion of the root expressing the reporter does grow out of the field of view, stop the FIJI macro, and pause the time lapse in VisiView. Use the “view live” option to manually reposition the stage. Then choose “resume acquisition” in the VisiView GUI and relaunch the FIJI macro. Tracking should resume as normal.

In the case that it is desirable to perform automated root tracking in the absence of a fluorescent reporter, there are published tools to accomplish this such as TipTracker (von Wangenheim et al., 2017a), which can be swapped for our FIJI macro.

#### Manual root tip tracking

For shorter-term time lapses, manually tracking the root tip is feasible. In our hands, the root tip grows out of the field of view roughly once per hour. As such, the time lapse will require frequent monitoring.

Using “Show Live”, position the stage such that the root tip is in the field of view. To maximize the amount of time between manual stage repositioning, it is ideal to position the stage such that the root tip is slightly off center and on the far side of the center of the field of view relative to the primary direction of growth. To initiate the time lapse, choose the “Sequence” option in the VisiView GUI instead of starting the time lapse using the macro code. When the root tip approaches the edge of the field of view, pause the time lapse acquisition using the “Pause Sequence” button. Then, select the “Show Live” button. Reposition the stage so that the root tip is again in the desired position. Then select, “Resume Sequence”. Repeat these steps as necessary until the desired time lapse is complete.

### Time lapse post-processing

After acquisition it is necessary to post-process the resulting time lapse in deconvolution and registration steps. We briefly describe how we perform these steps as they have been described elsewhere (for example see (Zhang et al., 2024)) and other options for deconvolution, such as AutoQuant X3, and registration could be applied. Deconvolution is necessary to remove optical distortion to improve resolution in the resulting image. The registration step will align different time frames in the x, y, and z dimensions in the time lapse such that the root does not move between time points. This step makes identifying cell-level dynamics easier both by eye and using automated tracking tools.

We perform deconvolution using Imaris software as implemented in the “Image Processing” menu. Individual experiments will require independent choice of settings based on the fluorophores and objectives used in each experiment, both of which are required input for the deconvolution algorithm. This step can take a long time and requires the system to have free memory that is at least double the size of the file being processed.

Despite the high performance of the Imaris software, we also found that the application would sometimes crash unexpectedly or produce a registered or deconvoluted file that was corrupted, i.e. having empty voxels in part of the image in one or more channels. Therefore, it is critical to save all intermediate files to ensure time-intensive processing steps do not need to be repeated. This creates additional storage considerations, as intermediate files will be large. Prepare a storage environment accordingly. We found that for short term storage, a large solid-state drive was ideal for handling intermediate files that are no longer necessary to maintain once the fully processed time lapse movie file is complete.

For smaller file sizes generated from time lapses of roots with a bright QC or columella marker, we found registration can readily be performed in FIJI using the “Correct 3d Drift” plugin. These smaller files may be generated from shorter time lapses or possibly longer time lapses with fewer acquisition channels or lower time resolution (< 10 Gb). Detailed instructions for the use of this plugin can be found here (Parslow et al., 2014).

For larger files (> 10 Gb), or for time lapses generated without a bright, point source marker such as a QC reporter, registration is better performed in Imaris. We find that this is most easily accomplished in a two-step process in which registration is first performed roughly by manually correcting the image reference frame. This is done in Imaris using the Reference Frame tool by positioning the reference coordinate system relative to the sample across time points. Aspects of the root such as nuclei can then be automatically segmented and tracked. The tracked elements can then be used to perform a secondary, fine-grained reference frame correction.

If desired, the unprocessed data exported by VisiView can also be used to analyze other aspects of the time lapse data such as the physical distance the sample grew during the time lapse. VisiView saves the time lapse in files containing an individual z stack per color, per time point as a “.stk” file. Each file contains metadata including stage position and acquisition time. This metadata can be accessed for an individual file with the Bio-Formats importer plugin for FIJI with the “Display metadata” option selected. Alternatively, Bio-Formats also exists as an R package - RBioFormats - which can be used to directly import metadata into R for computational purposes.

Data storage is a major consideration for long term time lapses. A time lapse of the full z depth in 3 colors with ten-minute resolution can generate roughly 5 Gb of data per hour. We find that a combination of high-capacity solid state drives and cloud storage are useful for transferring and warehousing the resulting datasets. In addition, the workstations used to run and analyze the data should have sufficient memory and storage to accommodate these files. See the materials section for the specifications of the workstation used for this study.

### Special experimental considerations

#### Contamination

Bacterial or fungal contamination will affect root growth and confound the experiment. Manipulating the media blanket creates opportunities to introduce contamination. Therefore, it is important to always work in a sterile environment and to ensure the forceps used to move the blanket are sterile. This can be accomplished by autoclaving the forceps and then submerging them in 70% ethanol prior to use. Immediately prior to use, move forceps into the sterile environment where work will be performed, remove from ethanol, and place on a sterile surface to allow the ethanol to evaporate.

#### Physical imperfections in media

Physical imperfections in the media will distort the light sheet and lead to banding artifacts in the resulting time lapse. This is unavoidable to an extent using this blanket method, which is necessary to constrain root movement in the z dimension. However, banding can be minimized by carefully manipulating the blanket to limit damaging it. If the blanket falls apart during transfer, discard it and use another. Small gaps between the blanket and the side wall of the chambered cover glass through which the light sheet passes can be addressed by pipetting a small amount of sterile and filtered ½ MS media with 2% low melt agarose at 30˚C to fill in any air pockets. This media should not come into contact with the seedling itself and should therefore not contribute to any temperature-related stress.

#### Mounting the seedlings without crushing the roots

During the mounting process it is possible to damage the root tips. To ensure that roots are mounted without damage it is advisable to visually inspect mounted roots under a dissecting microscope. Damaged roots will be more opaque than undamaged roots or their shape will be distorted. Damaged roots should not be used for imaging.

#### Seedling placement

As the sheet travels through the media signal intensity is lost. Therefore, positioning the root closer to the sheet emission point in the x dimension will improve imaging quality. It is ideal to keep this in mind when mounting samples. In addition, it is critical that there should be no objects between the imaged seedling and the light sheet emission point to prevent any potential shadows or dark points in the image.

## Results

**Figure 4:**
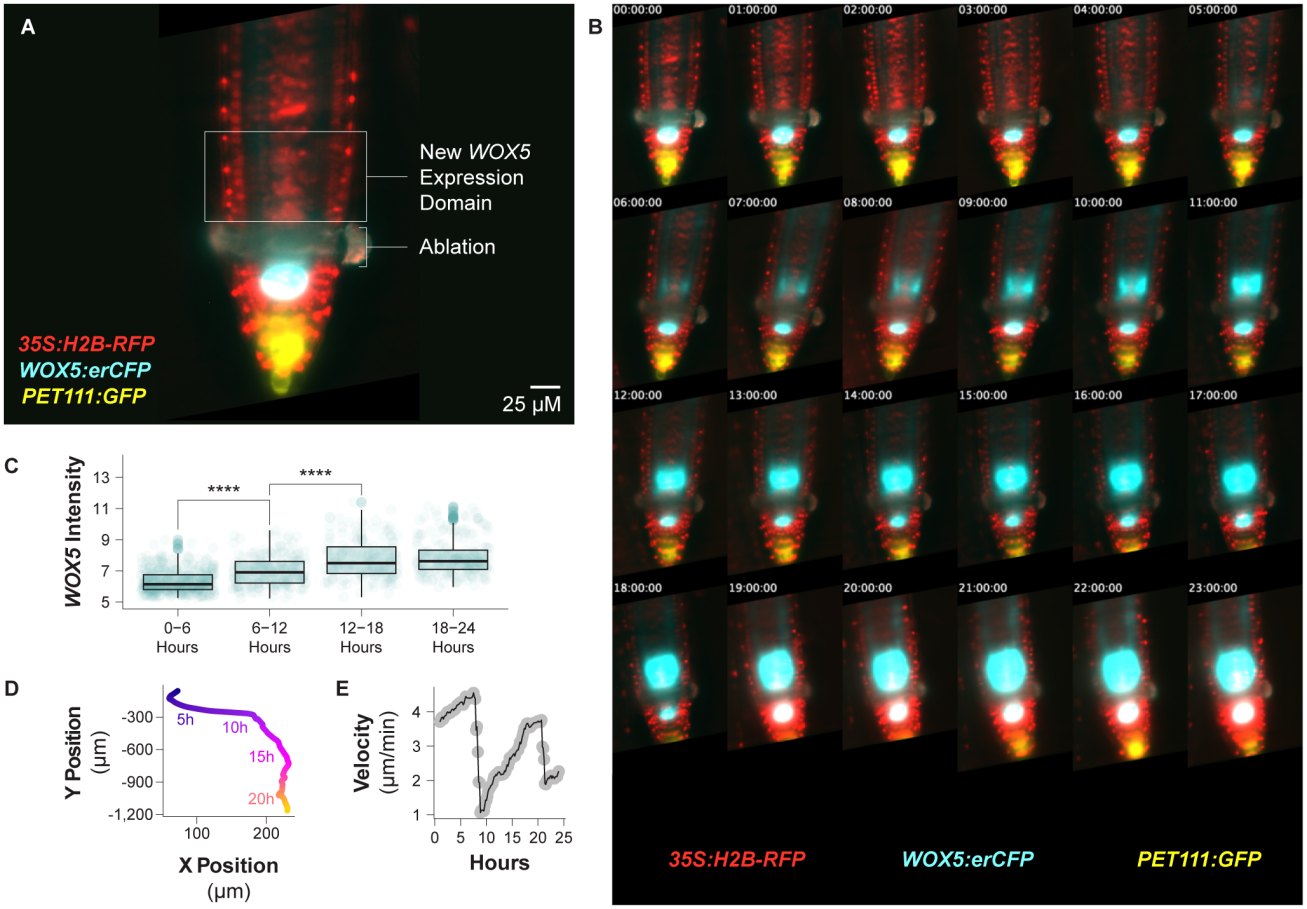
Continuous imaging of a regenerating root over 24 hours. **(A)** A median optical section of an injured root expressing three different fluorescent reporters immediately following laser ablation. The ablation site and future *WOX5* expression domain are annotated. The root expresses *35S:H2B-RFP*, *WOX5:erCFP*, and *PET111:GFP*. **(B)** Median slices of that same root over 24 hours, here shown once per hour. Over that time course the new *WOX5:erCFP* expression domain is established. **(C)** A boxplot showing *WOX5:erCFP* intensity on a per cell basis binned over six-hour time periods within the new *WOX5* expression domain. Expression levels for this reporter significantly increase over the first two time periods (**** indicates p value <= 4e-7 by the Student’s T test). **(D)** The relative position of the QC over time during the entire time lapse acquisition. Time points are color coded. **(E)** The velocity of the root tip over the course of the time lapse acquisition.

Previously we have used this time lapse microscopy system to study cell cycle kinetics during normal growth and regeneration in Arabidopsis roots, where we observed good concordance between cell cycle behaviors as measured by light sheet microscopy and single cell RNA-seq (Lee et al., 2025). Here, we demonstrate the utility of this light sheet imaging system by performing a long-term time lapse on a root expressing three fluorescent protein fusion constructs: *35s:H2B-RFP*, *WOX5:erCFP*, and *PET111:YFP* (**Figure 4A**). We also chose to demonstrate that the imaging experimental design, while deviating from standard plant growth conditions, does not interfere with core developmental processes by performing this time lapse on a regenerating root. As described in (Lee et al., 2025), we injured the root using a 2-photon laser in an ROI positioned anticlinally to the growth vector of the root in three z planes on a Zeiss LSM 880. We then generated a 24-hour time lapse in three colors with ten-minute time resolution. At each time point we captured the full z dimension of the root with slices spaced 1.5 microns apart.

Over the course of the 24 hour time lapse, the expression domain of the *WOX5* reporter shifts to the position where the new QC will form (**Figure 4B**, **Supplementary Video 1**), consistent with prior observations (Sena et al., 2009; Efroni et al., 2016; Lee et al., 2025). With this technique, we confirm previous reports (Rahni et al., 2024; Lee et al., 2025) that *WOX5* expression in this new domain begins between four and five hours post ablation (**Figure 4B**). Measured on a per cell basis and binned into 6-hour time periods, this signal increases significantly over the first 18 hours of the time lapse (**Figure 4C**). The establishment of a new *WOX5* domain proximal to the wound site demonstrates that root tip regeneration occurs unimpeded in this imaging system. This agrees with our previous results using the TILT imaging system to study root tip regeneration (Lee et al., 2025). One advantage of this system is the fine time resolution, which allows us to capture a larger subset of mitotic events, which are often only visible for 20 minutes or less, than other imaging techniques that require lower time resolution. This makes it possible for us to confidently correlate cell divisions with changes in marker expression domain. It has previously been shown that the *WOX5* expression domain’s position is refined during regeneration, both to mark the new QC and in coordination with the formation of a proximal and distal domain, where *WOX5* is expressed in the proximal domain (Efroni et al., 2016). Here we can see that the *WOX5* expression domain is delineated in part by the division of cells located distally within the *WOX5* expression domain (**Supplementary Figure 1**).

To demonstrate normal root growth in this system, we extracted the stage position metadata to track root position over time (**Figure 4D**). The root moved predominantly in the y dimension, which is parallel to the light sheet, and remained close enough to the cover slip to remain within the working distance of the objective for 24 hours. We calculated the velocity of the root tip over time and found the velocity rose and fell periodically over the 24 hour time lapse (**Figure 4E**), consistent with prior reports describing growth in unconstrained roots (Yazdanbakhsh and Fisahn, 2010). This indicates that, despite the need to constrain root growth in the x and z dimensions (against the gravity vector), the root growth is largely unperturbed. In addition, we observe normal developmental milestones such as the correct establishment of root hairs in the guide root.

## Discussion

Time lapse imaging of plants is a powerful tool to determine how organs develop and how individual cells contribute to that process over time. Establishment of new imaging systems in a lab can be costly and may require significant technical expertise. Here, we describe the application of a relatively low cost, easy to use light sheet microscopy system as applied to time lapse imaging of plant roots. We demonstrate the capability of this system to automatically track an injured root tip over 24 hours, during which time that root undergoes regeneration. We monitor the expression of a cell fate marker gene on a per cell basis over time to establish the ability of tracking cellular behaviors during complex developmental events.

Tools such as this can be applied to a diverse set of biological phenomena such as cell cycle regulation, cell identity transitions, organ patterning and plant-environment interactions. While the protocol presented here is optimized for imaging root tips, which require specific sample mounting strategies based on organ geometry and growth patterns, a low footprint light sheet imaging system such as this will be useful to plant biologists studying other organs and systems as well. However, we note this system is not optimal for specifically studying roots response to gravity, as the mounting strategy required for this configuration prevents the root from growing towards the gravity vector. In particular, this system could be applied to imaging cells and structures located deep within a tissue that are difficult to visualize with a confocal microscopy approach. For instance, imaging the early events during callus formation, which can derive from cells deep within an explant (Ikeuchi et al., 2013), might best be imaged with a system such as this. Additionally, the low phototoxicity generated by light sheet imaging renders this system appropriate for time lapse imaging of long processes, like callus formation.

Increasing access to light sheet imaging techniques with relatively inexpensive and low-footprint microscopes is likely to have benefits outside of model systems like Arabidopsis. Looking forward, it could be advantageous to the plant biology community to develop similar protocols to study other organs and other species. While the protocol presented here uses fluorescence intensity of a reporter to track the growing root tip, other shape-based tracking algorithms exist (von Wangenheim et al., 2017a; Buckner et al., 2019; Goh et al., 2023) and could be applied here to expand the kinds of plants and even Arabidopsis lines that can be studied via long term time lapse microscopy. Additionally, the imaging system presented here is compatible with microfluidics imaging slides, so another exciting future direction could be adapting systems like the RootChip (Grossmann et al., 2011) for use with this style of light sheet microscope without requiring a custom microscopy system. Finally, lack of light exposure over exceptionally long imaging times may cause additional stress for the growing seedling. Therefore, another future direction worth considering is adaptation of a previously published lighting system appropriate for light sheet microscopy (Baesso et al., 2018).

Overall, light sheet microscopy is a powerful tool for studying plant cell and developmental biology. Expanding access to this imaging modality will facilitate a growing body of excellent work using microscopy to study plant biology.

## Data Availability

The raw data supporting the conclusions of this article will be made available by the authors, without undue reservation.

## Supplementary material

Supplementary Figure 1 Tracked cell division that shapes the WOX5 domain. A cell and the daughters of its division are shown with white arrows. The cell divides at an angle that marks the boundary of the new *WOX5* domain.

Supplementary Figure 2 Execution of VisiView Macro. A screen shot of the implementation of the tracking script in VisiView. The red arrow at the top of the screen points out the “Macros” menu where the user can find the option to open the provided macro. The settings can be updated per run in the editing window at the bottom of the screen where indicated by the comments in the code. When ready, the user run the macro with the “Run” button to the right of the editor, pointed out with another red arrow.

Supplementary Figure 3 Execution of FIJI Macro. A screen shot of the implementation of the provided FIJI macro used for tracking. This view of the macro can be established by opening the provided macro using the “Plugins” menu (red arrow at the top of the screen shot) and selecting “Macros > Edit”. The macro can be edited as necessary for the conditions of the particular run where indicated by the code comments. The macro can then be executed by selecting the “Run” button (red arrow at the bottom left of the screen shot). Once the macro is executed, pop-up windows will open asking the user to specify the time between acquisitions, the run directory, and the channel to use for tracking.

Supplementary Video 1 Regeneration time lapse. This Supplementary video corresponds to **Figure 4B** and shows a median optical section of a regenerating root expressing *35S:H2B-RFP*, *WOX5:erCFP*, and *PET111:GFP*.

Supplementary Video 2 Mounting procedure. The Supplementary video demonstrates the mounting procedure described in **Figure 1**.

## References

[B1] Baesso P. Randall R. S. Sena G. (2018). Light sheet fluorescence microscopy optimized for long-term imaging of Arabidopsis root development. Methods Mol. Biol. 1761, 145–163. doi: 10.1007/978-1-4939-7747-5_11 29525955

[B2] Buckner E. Madison I. Chou H. Matthiadis A. Melvin C. E. Sozzani R. . (2019). Automated imaging, tracking, and analytics pipeline for differentiating environmental effects on root meristematic cell division. Front. Plant Sci. 10. doi: 10.3389/fpls.2019.01487 PMC6877711 31803217

[B3] Candeo A. Doccula F. G. Valentini G. Bassi A. Costa A. (2017). Light sheet fluorescence microscopy quantifies calcium oscillations in root hairs of Arabidopsis thaliana. Plant Cell Physiol. 58, 1161–1172. doi: 10.1093/pcp/pcx045 28379562 PMC6383626

[B4] Combs C. A. Shroff H. (2017). Fluorescence microscopy: A concise guide to current imaging methods. Curr. Protoc. Neurosci. 79, 2.1.1–2.1.25. doi: 10.1002/cpns.29 28398640

[B5] Dong J. MacAlister C. A. Bergmann D. C. (2009). BASL controls asymmetric cell division in arabidopsis. Cell 137, 1320–1330. doi: 10.1016/J.CELL.2009.04.018 19523675 PMC4105981

[B6] Efroni I. Mello A. Nawy T. Ip P.-L. Rahni R. DelRose N. . (2016). Root regeneration triggers an embryo-like sequence guided by hormonal interactions. Cell 165, 1721–1733. doi: 10.1016/j.cell.2016.04.046 27212234 PMC4912400

[B7] Elliott A. D. (2020). Confocal microscopy: Principles and modern practices. Curr. Protoc. Cytom. 92, e68. doi: 10.1002/cpcy.68 31876974 PMC6961134

[B8] Goh T. Song Y. Yonekura T. Obushi N. Den Z. Imizu K. . (2023). In-depth quantification of cell division and elongation dynamics at the tip of growing Arabidopsis roots using 4D microscopy, AI-assisted image processing and data sonification. Plant Cell Physiol. 64, 1262–1278. doi: 10.1093/pcp/pcad105 37861079 PMC10700013

[B9] Grossmann G. Guo W.-J. Ehrhardt D. W. Frommer W. B. Sit R. V. Quake S. R. . (2011). The RootChip: an integrated microfluidic chip for plant science. Plant Cell 23, 4234–4240. doi: 10.1105/tpc.111.092577 22186371 PMC3269862

[B10] Hobson C. M. Guo M. Vishwasrao H. D. Wu Y. Shroff H. Chew T.-L. (2022). Practical considerations for quantitative light sheet fluorescence microscopy. Nat. Methods 19, 1–12. doi: 10.1038/s41592-022-01632-x 36266466

[B11] Ikeuchi M. Sugimoto K. Iwase A. (2013). Plant callus: mechanisms of induction and repression. Plant Cell 25, 3159–3173. doi: 10.1105/tpc.113.116053 24076977 PMC3809525

[B12] Lee L. R. Guillotin B. Rahni R. Hutchison C. Desvoyes B. Gutiérrez C. . (2025). Glutathione accelerates the cell cycle and cellular reprogramming in plant regeneration. Dev. Cell 60, 1153–1167.e6. doi: 10.1016/j.devcel.2024.12.019 39755116 PMC12278113

[B13] Li X.-M. Jenke H. Strauss S. Wang Y. Bhatia N. Kierzkowski D. . (2024). Age-associated growth control modifies leaf proximodistal symmetry and enabled leaf shape diversification. Curr. Biol. 34, 4547–4558.e9. doi: 10.1016/j.cub.2024.07.068 39216485

[B14] Müller B. Sheen J. (2008). Cytokinin and auxin interaction in root stem-cell specification during early embryogenesis. Nature 453, 1094–1097. doi: 10.1038/nature06943 18463635 PMC2601652

[B15] North A. J. (2006). Seeing is believing? A beginners’ guide to practical pitfalls in image acquisition. J. Cell Biol. 172, 9–18. doi: 10.1083/jcb.200507103 16390995 PMC2063524

[B16] Ovečka M. Sojka J. Tichá M. Komis G. Basheer J. Marchetti C. . (2022). Imaging plant cells and organs with light-sheet and super-resolution microscopy. Plant Physiol. 188, 683–702. doi: 10.1093/plphys/kiab349 35235660 PMC8825356

[B17] Ovečka M. von Wangenheim D. Tomančák P. Šamajová O. Komis G. Šamaj J. (2018). Multiscale imaging of plant development by light-sheet fluorescence microscopy. Nat. Plants 4, 639–650. doi: 10.1038/s41477-018-0238-2 30185982

[B18] Parslow A. Cardona A. Bryson-Richardson R. J. (2014). Sample drift correction following 4D confocal time-lapse imaging. J. Vis. Exp. (86), e51086. doi: 10.3791/51086 PMC4166950 24747942

[B19] Power R. M. Huisken J. (2017). A guide to light-sheet fluorescence microscopy for multiscale imaging. Nat. Methods 14, 360–373. doi: 10.1038/nmeth.4224 28362435

[B20] Rahni R. Guillotin B. Lee L. R. Birnbaum K. D. (2024). A temporal map of division, chromatin modification, and identity specification in the regenerating root. bioRxiv 2024.01.09.574680. doi: 10.1101/2024.01.09.574680

[B21] Sena G. Wang X. Liu H.-Y. Hofhuis H. Birnbaum K. D. (2009). Organ regeneration does not require a functional stem cell niche in plants. Nature 457, 1150–1153. doi: 10.1038/nature07597 19182776 PMC2649681

[B22] Toyota M. Spencer D. Sawai-Toyota S. Jiaqi W. Zhang T. Koo A. J. . (2018). Glutamate triggers long-distance, calcium-based plant defense signaling. Science 361, 1112–1115. doi: 10.1126/science.aat7744 30213912

[B23] von Wangenheim D. Hauschild R. Fendrych M. Barone V. Benková E. Friml J. (2017a). Live tracking of moving samples in confocal microscopy for vertically grown roots. Elife 6, e26792. doi: 10.7554/eLife.26792 28628006 PMC5498147

[B24] von Wangenheim D. Hauschild R. Friml J. (2017b). Light sheet fluorescence microscopy of plant roots growing on the surface of a gel. J. Vis. Exp 119, e55044. doi: 10.3791/55044 PMC5352271 28190052

[B25] Winter C. M. Szekely P. Popov V. Belcher H. Carter R. Jones M. . (2024). SHR and SCR coordinate root patterning and growth early in the cell cycle. Nature 626, 611–616. doi: 10.1038/s41586-023-06971-z 38297119 PMC10866714

[B26] Yazdanbakhsh N. Fisahn J. (2010). Analysis of Arabidopsis thaliana root growth kinetics with high temporal and spatial resolution. Ann. Bot. 105, 783–791. doi: 10.1093/aob/mcq048 20421235 PMC2859919

[B27] Zhang D. Cleveland A. H. Krimitza E. Han K. Yi C. Stout A. L. . (2024). Spatial analysis of tissue immunity and vascularity by light sheet fluorescence microscopy. Nat. Protoc. 19, 1053–1082. doi: 10.1038/s41596-023-00941-5 38212641 PMC12276897

