# Supplementary figures and images for: A long term time lapse microscopy technique for Arabidopsis roots
Source: Front Plant Sci. 2025 Jun 9;16:1601397. doi: 10.3389/fpls.2025.1601397 (PMC12183276; doi:10.3389/fpls.2025.1601397)

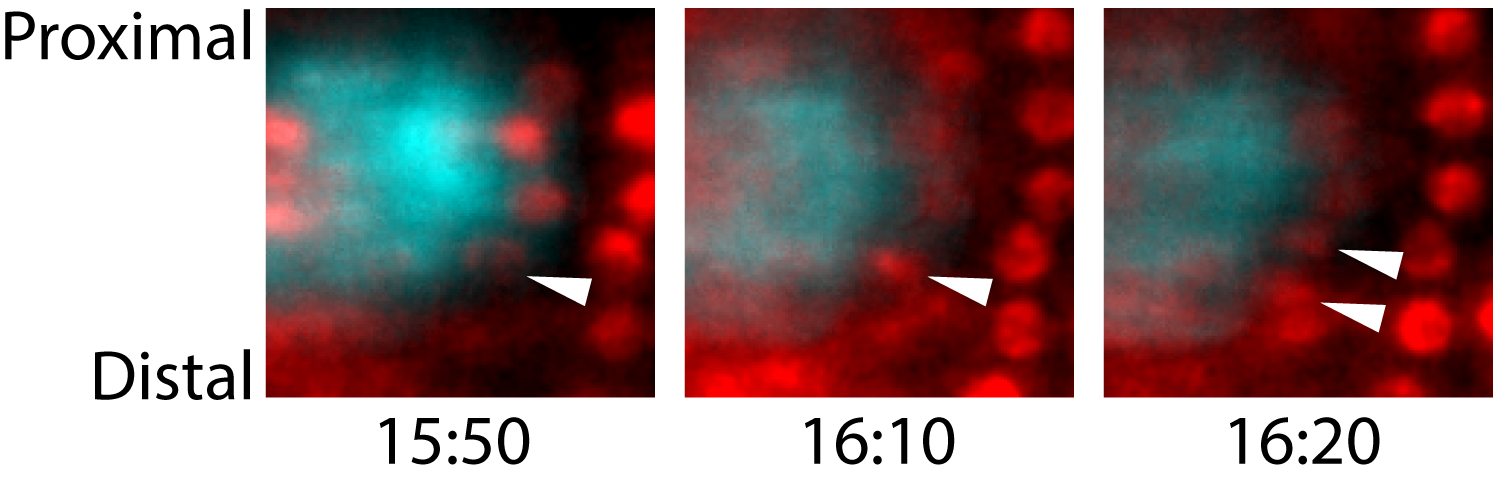

Supplement: Supplementary Figure 1 — Tracked cell division that shapes the WOX5 domain. A cell and the daughters of its division are shown with white arrows. The cell divides at an angle that marks the boundary of the new WOX5 domain. [file Image1.tiff]

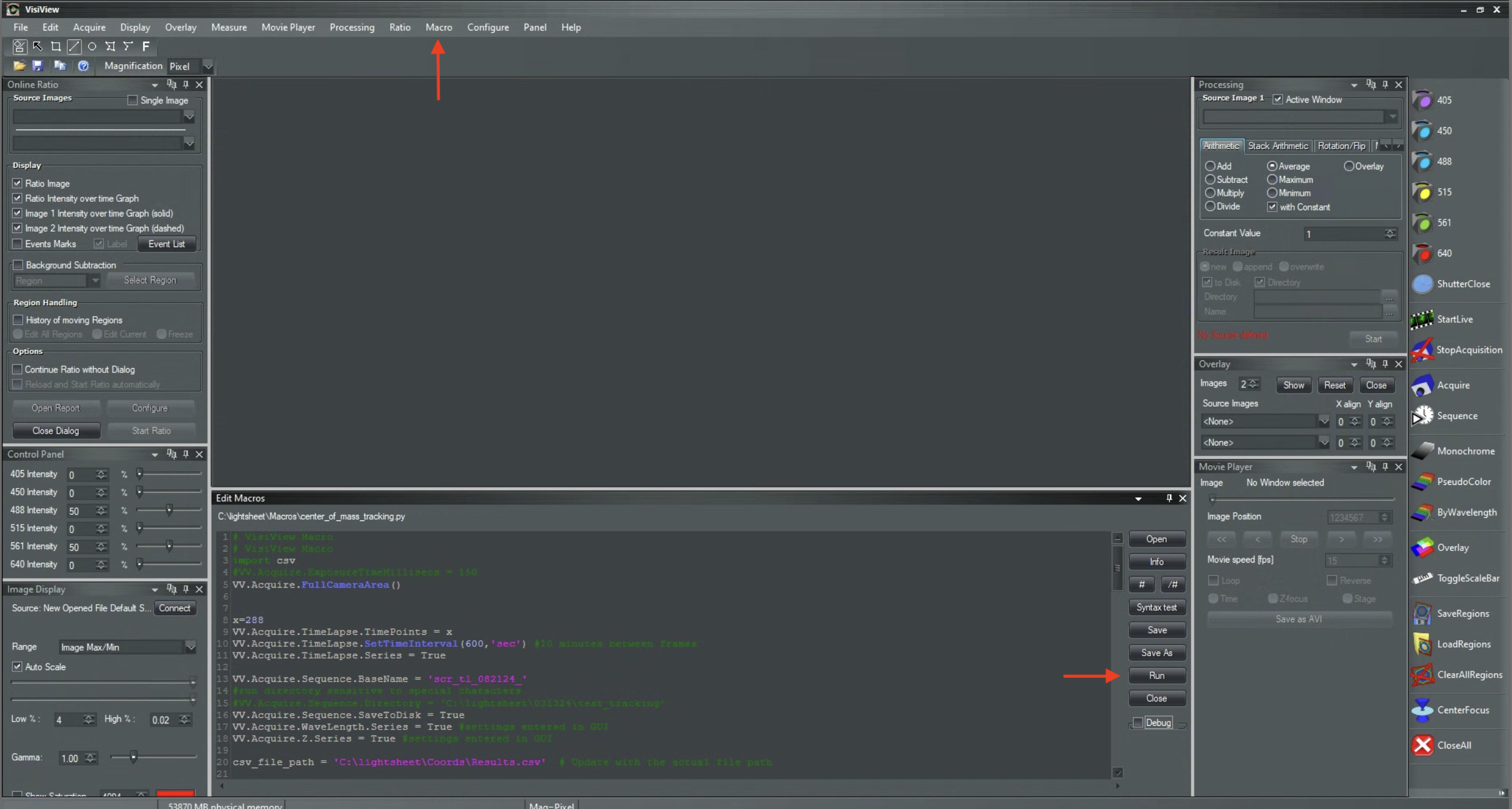

Supplement: Supplementary Figure 2 — Execution of VisiView Macro. A screen shot of the implementation of the tracking script in VisiView. The red arrow at the top of the screen points out the “Macros” menu where the user can find the option to open the provided macro. The settings can be updated per run in the editing window at the bottom of the screen where indicated by the comments in the code. When ready, the user run the macro with the “Run” button to the right of the editor, pointed out with another red arrow. [file Image2.png]

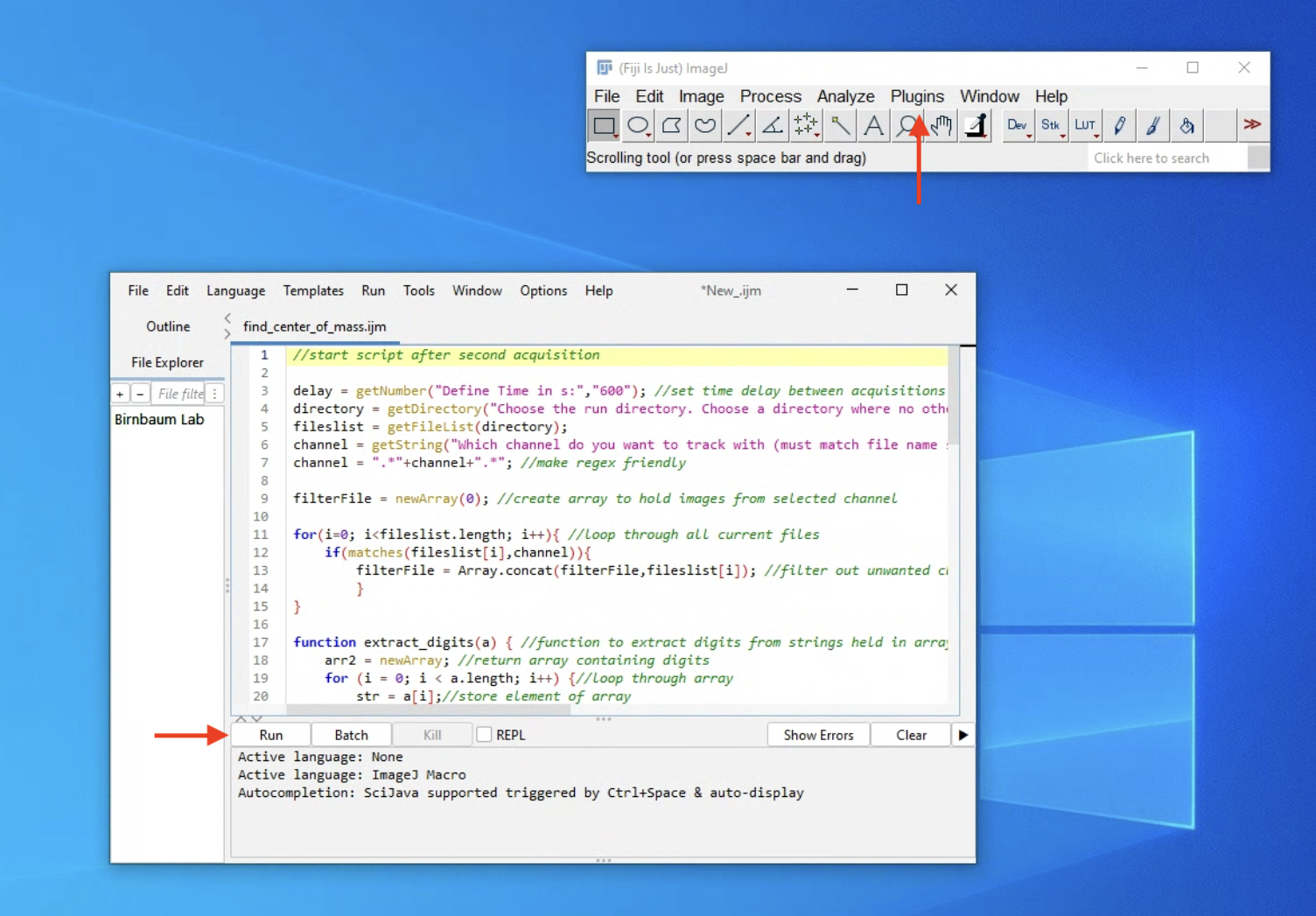

Supplement: Supplementary Figure 3 — Execution of FIJI Macro. A screen shot of the implementation of the provided FIJI macro used for tracking. This view of the macro can be established by opening the provided macro using the “Plugins” menu (red arrow at the top of the screen shot) and selecting “Macros > Edit”. The macro can be edited as necessary for the conditions of the particular run where indicated by the code comments. The macro can then be executed by selecting the “Run” button (red arrow at the bottom left of the screen shot). Once the macro is executed, pop-up windows will open asking the user to specify the time between acquisitions, the run directory, and the channel to use for tracking. [file Image3.png]
